# Telehealth in oncology: a cost analysis to evaluate the financial impact of implementing regional trial hubs within a phase 3 cancer clinical trial

**DOI:** 10.1111/imj.16292

**Published:** 2023-12-21

**Authors:** Marliese Alexander, Ian Collins, Patrick Abraham, Craig Underhill, Sam Harris, Javier Torres, Sharad Sharma, Benjamin Solomon, An Tran‐Duy, Kate Burbury

**Affiliations:** ^1^ Pharmacy Department Peter MacCallum Cancer Centre Melbourne Victoria Australia; ^2^ Sir Peter MacCallum Department of Oncology The University of Melbourne Melbourne Victoria Australia; ^3^ Victorian Comprehensive Cancer Centre Melbourne Victoria Australia; ^4^ Deakin University Melbourne Victoria Australia; ^5^ Centre for Health Policy, Melbourne School of Population and Global Health The University of Melbourne Melbourne Victoria Australia; ^6^ Border Medical Oncology and Haematology Research Unit Albury Wodonga Regional Cancer Centre Albury Wodonga New South Wales Australia; ^7^ Rural Medical School University of New South Wales Sydney New South Wales Australia; ^8^ Bendigo Cancer Centre Bendigo Health Bendigo Victoria Australia; ^9^ Peter Copulos Cancer and Wellness Centre Goulburn Valley Health Shepparton Victoria Australia; ^10^ Shepparton Clinical School The University of Melbourne Shepparton Victoria Australia; ^11^ Ballarat Regional Integrated Cancer Centre Grampians Health Ballarat Victoria Australia; ^12^ Department of Medical Oncology Peter MacCallum Cancer Centre Melbourne Victoria Australia; ^13^ Department of Haematology Peter MacCallum Cancer Centre Melbourne Victoria Australia

**Keywords:** cost analysis, teletrial, patient cost, cancer, clinical trial

## Abstract

This cost analysis, from a societal perspective, compared the cost difference of a networked teletrial model (NTTM) with four regional hubs versus conventional trial operation at a single metropolitan specialist centre. The Australian phase 3 cancer interventional randomised controlled trial included 152 of 328 regional participants (patient enrolment 2018–2021; 6‐month primary end point). The NTTM significantly reduced (AU$2155 per patient) patient travel cost and time and lost productivity.

Virtual connectivity utilising telehealth (TH) is an accepted and widely implemented strategy to improve access to health care services. The networked teletrial model (NTTM) is an emerging paradigm of trial conduct incorporating digital technologies, in particular TH, to improve clinical trial access, participation and retention for patients living in regional and rural areas. We aimed to conduct a cost analysis to evaluate the financial impact of implementing the NTTM to conduct a phase 3 clinical trial for patients with cancer. The cost analysis was undertaken from the patient's perspective, and considered travel cost and time and productivity loss.

The trial, TARGET‐TP (Targeted Thromboprophylaxis in Ambulatory Patients Receiving Anticancer Therapies), was a randomised controlled trial assessing the safety and efficacy of biomarker‐driven risk‐directed thromboprophylaxis for patients receiving anticancer therapies (ACTRN12618000811202).^1^ Adults commencing systemic anticancer therapies for lung or gastrointestinal cancers without contraindications to pharmacologic thromboprophylaxis were enrolled from June 2018 to July 2021; the last follow‐up was December 2021. The trial was conducted using the NTTM.^2^, ^3^ The primary end point follow‐up period was 6 months, with all participants, regardless of cancer type or randomisation cohort scheduled for five follow‐up visits in this period.

This cost analysis compared scenarios to derive the potential direct and indirect cost difference when utilising a strategy that expanded the trial network for the purposes of trial conduct, which allowed patients to attend their local regional centre, as opposed to the metropolitan primary site. This expansion to regional hubs retained the governance, ethics, pharmacovigilance and trial conduct accountability under the responsibility of the primary trial team. The trial recieved Human Research Ethics Committee (HREC) approval HREC/18/PMCC/36 with substudy approval HREC/44490/PMCC‐2019.

There were three components for this analysis: cost per return trip, time per return trip (including wait times) and productivity cost associated with time loss because of waiting and travel. These three components were estimated for the actual events (regional/rural patients at hubs) and the hypothetical scenario (all regional patients attending the metropolitan site instead of their local hub; all metropolitan patients attending the metropolitan site as per trial conduct). For each patient, travel distance and times were estimated using the Google Maps Directions function, as recommended by the Victorian Patient Transport Assistance Scheme.^4^ The shortest route was chosen, and it was assumed that patients travelled by car. Distances were measured by patients' residential postcode to the metropolitan site and, where relevant, to the specified regional hub site. Travel costs were estimated using the rate of 0.72 cents per kilometre, from the Australian Tax Office cents per kilometre method for each return trip.^5^ A parking fee was assumed to incur only for patients who visited the metropolitan site, with a flat rate of AU$30 per visit based on an informed assumption of an average parking time of 5–6 h (factoring cancer treatment and trial consultation, usually coordinated together for patient convenience) per visit at standard (nonconcession) parking rates. Productivity loss estimates were determined by summing the time travelled in the return trip and the average time waiting at each facility of 50 min. To determine the productivity cost because of travel, the average human capital earnings of AU$36/h^6^ was universally applied to all patients because we did not have direct data on wages or indirect data on employment status (employed or unemployed), category of job (part‐ or full‐time) and types of job (e.g. manual or office‐based). The total costs reflected travel cost plus productivity cost associated with travel and wait time.

The trial enrolled 328 patients, including 152 (46%) from four regional centres located 119–325 km from the primary metropolitan site. The median age was 65 years (range, 30–88 years), 54%/46% were male/female and 92% were White/Caucasian. Patient characteristics (age, sex, new vs recurrent cancer, type of cancer treatment), described in full in the primary outcomes paper,^1^ were balanced between metropolitan and regional sites, other than a higher proportion of regional/rural participants with gastrointestinal cancers compared with the metropolitan site; reflective of referral patterns and not considered relevant to this economic report. There was no difference in trial conduct between the metropolitan and regional sites, including visit adherence, data quality, safety reporting and regulatory compliance.

Among regional patients, average differences in travel cost from patient home to regional centre (actual) versus patient home to metropolitan cancer centre (theoretical) were AU$210, AU$408, AU$286 and AU$192 per patient for each of the four regional centres. The average differences in time to attend trial appointments at regional versus metropolitan centres were 161, 314, 225 and 145 min per patient, equivalent to average productivity loss (time–cost difference) of AU$97, AU$188, AU$135 and AU$87 per patient. The total cost difference per patient per trial visit (travel cost and productivity losses) was calculated at AU$307, AU$596, AU$421 and AU$279 for each regional centre. If all regional patients (*n* = 152) were required to attend the metropolitan specialist centre for trial participation, instead of regional hubs, the total cost difference was calculated at AU$327 524 for all trial visits (five visits in the primary follow‐up period) or AU$2155 per regional trial participant (Table 1).

**Table 1:** Travel distance, cost and time and productivity cost associated with travel to a metropolitan trial hub compared with a regional hub as part of the NTTM

Site	Metropolitan (*n* = 176)	Regional 2 (*n* = 74)	Regional 1 (*n* = 61)	Regional 4 (*n* = 12)	Regional 3 (*n* = 5)
Distance (km)					
Local to metropolitan site	0	151	325	190	118
Travel cost (AU$)					
Home to local site	$55.21	$63.26	$91.91	$46.74	$28.83
Home to metropolitan site	$55.21	$243.06	$470.15	$303.00	$190.94
Parking at metropolitan site	$30.00	$30.00	$30.00	$30.00	$30.00
Cost difference	$0	$209.80	$408.23	$286.26	$192.12
Travel time (min)					
Home to local site	125.85	120.00	146.13	104.50	90.80
Home to metropolitan site	125.85	281.16	459.61	329.33	235.60
Time difference	0	161.16	313.48	224.83	144.80
Productivity cost associated with time loss					
Home to local site	$75.51	$72.00	$87.68	$62.70	$54.48
Home to metropolitan site	$75.51	$168.70	$275.76	$197.60	$141.36
Time–cost difference	‐	$96.70	$188.09	$134.90	$86.88
Total per patient per visit					
Care at local site	$160.72	$135.26	$179.59	$109.44	$83.31
Care at metropolitan site	$160.72	$441.76	$775.91	$530.60	$362.30
Cost difference	$0	$306.49	$596.32	$421.16	$279.00
Total per patient over trial†					
Care at local site	$803.60	$676.30	$897.95	$547.20	$416.55
Care at metropolitan site	$803.60	$2208.80	$3879.55	$2653.00	$1811.50
Cost difference	$0	$1532.45	$2981.60	$2105.80	$1395.00
Total per trial site over trial‡	$0	$113 403.12	$181 877.28	$25 269.60	$6975.00
Total for all sites over trial§					$327 524.88

† Multiplies per patient visit cost by five visits included in the trial's primary follow‐up period.

‡ Multiplies total per‐patient costs by number of patients enrolled at each site.

§ Sum of total patient costs at all sites.

NTTM, networked teletrial model.

## Discussion

Implementation of the NTTM is not only a strategy to increase regional and rural trial participation and improve outcomes through access to high‐level care and novel therapies but it also values patient time and minimises the financial impact of trial participation. Although this analysis is limited to patient costs, and modelled in a single clinical trial, it demonstrates significant potential patient savings should NTTM be routine across cancer trials and centres globally.

The recent post–coronavirus disease 2019 (COVID‐19) pandemic literature has exploded with papers describing the implementation and outcomes of NTTM across different countries and disease/treatment settings as TH has become embedded into usual care. Within the Australian context, we can identify papers on implementation,^2^, ^3^, ^7^, ^8^, ^9^ trial recruitment^10^ and patient experience.^11^ To our knowledge, whereas cost analysis for TH in oncology general practice has previously been reported,^12^ this is the first Australian paper to report cost‐analysis for NTTM in oncology from the patient perspective.

This analysis was undertaken to provide an estimate of patient costs in an individual trial. Estimates are considered to be highly conservative given the use of direct travel cost/time calculations assuming a same‐day two‐way trip. In reality, patients may stay overnight, incurring additional accommodation costs and productivity loss. Moreover, even assuming same‐day two‐way trips, whereas the productivity loss in our analysis reflects actual time travelling and attending appointments, patients (and caregivers, not included in this analysis) will usually experience productivity loss for an entire day, i.e. required to take a full day off work. Although these details were not captured in the trial, limiting the current analysis to estimation at the base/conservative level for the patient only, it is highly reasonable to conclude that these figures likely underrepresent true cost‐savings of the NTTM. We highlight the relative simplicity and short‐term follow‐up (five visits over 6 months) of the TARGET‐TP trial compared with most interventional clinical trials, which would achieve even further savings multiplied out over increased visits and time periods.

One limitation in our estimation of the productivity cost associated with patients' time loss using the Human Capital Approach^13^ is that we used the same average wage for all participants because of the lack of direct data on wages or indirect data on employment status and types of job. In addition, we did not have data on informal care, such as volunteer and caregiver time, who assist the participants in travel and unpaid domestic work during home absence. Therefore, the actual productivity costs, including all components, could be much higher than our estimate. Another limitation relates to the assumption of a flat parking fee of AU$30 per visit to the metropolitan site, as there were no data on concession card holders or actual parking time or parking rate. As we used the parking rate at the metropolitan public hospital, which is cheaper compared with other parking options in the vicinity, this might offset the overestimation when no concession fees were considered. Therefore, we speculated that our estimates of the parking fee were not biased.

Health services or trial operating costs were not factored in this analysis, which for this trial were similar irrespective of enrolment site (regional or metropolitan). However, we acknowledge that this may not be the situation for all trials conducted under the NTTM with the potential for increased operational costs in the remote management of regional patients (e.g. medicine supply and health care professional and administration costs both at regional and primary sites).

This cost analysis of the phase 3 Australian multisite TARGET‐TP trial in patients with cancer has demonstrated a significant reduction in patient travel cost and time and lost productivity. A mean cost‐saving of AU$2155 per patient was achieved by implementing the NTTM allowing regional and rural patients to receive care at local hub centres, rather than travelling to a metropolitan centre. Although data limitations necessitated several assumptions and estimations, these represent the most conservative scenarios and give confidence to the conclusion that NTTM reduces direct patient costs. The first analysis of its kind in the Australian context, these findings will encourage broader adoption of the NTTM to support greater participation of regional and rural patients in clinical trials.
